# Supplementary material for: Are carbon emissions trading and green financial instruments synergistic? -Comprehensive quantitative research based on content analysis
Source: PLoS One. 2024 Mar 7;19(3):e0298601. doi: 10.1371/journal.pone.0298601 (PMC10919601; doi:10.1371/journal.pone.0298601)
Supplement: S1 Data — (ZIP) [file pone.0298601.s001.zip › Data/地级市Z_F工作报告.zip!/║■▒▒/╥╦▓2╩╨/└┤╫╘╣1⁄2╓┌║┼í╛┬φ┐╦╩2╛▌═°í┐_2013.docx]

各位代表：

　　现在，我代表市人民政府向大会报告工作，请予审议，并请各位政协委员和列席人员提出意见。

　　一、2012年工作回顾

　　2012年，面对复杂严峻的国内外经济形势，市政府在市委正确领导下，在市人大和市政协监督支持下，紧紧依靠全市人民，深入践行科学发展观，突出稳中求进、好中求快，应急谋远，克难奋进，团结实干，全市经济社会继续保持又好又快的发展态势，主要经济指标好于全国全省平均水平。预计生产总值超过2500亿元，同比增长13%左右。地方公共财政预算收入153.2亿元，增长34.4%。预计全社会固定资产投资1600亿元，增长34.2%；城镇居民人均可支配收入1.87万元，农村居民人均纯收入8043元，均增长14%；外贸出口17.3亿美元，增长0.8%；社会消费品零售总额733亿元，增长16%。除外贸出口和社会消费品零售总额指标外，市五届人大一次会议确定的目标任务全部完成。

　　（一）强化政策扶持，经济活力进一步增强。深入开展“迎创推”主题实践活动，拓宽发展思路，创新体制机制，致力服务市场主体。加快实施中小企业成长工程，净增规模以上工业企业145家，达到1010家；新增限额以上商贸流通企业300家以上，突破1650家；3739家个体工商户转型升级为企业。新增各类市场主体5.1万户，新增注册资本额同比增长77.8%。出台九条财政扶持措施，促进城区经济加快发展。出台“飞地工业”发展政策，支持各县市区到宜昌高新区发展工业。出台扶持奖励措施，鼓励企业进入“新三板”和其他场外市场挂牌上市。2家企业在天津股权交易所挂牌，4家企业进入上市报备辅导期。财投公司、城投公司分别发行债券15亿元和10亿元，兴发股份通过增发再融资13.5亿元。完善中小企业融资担保体系，融资担保机构增资12.3亿元。深化银政企对接，银行业金融机构存款余额2010.3亿元，贷款余额1455.3亿元。引导金融机构加大支持中小微企业力度，中小微企业贷款占全部企业贷款比重达到59.8%。开展营业税改征增值税试点，近8000家企业受益。

　　（二）狠抓项目建设，发展后劲进一步夯实。扎实开展“重大项目建设年”活动，统筹推进产业、基础设施和民生项目建设。全市施工亿元以上项目640个，增加284个，投资额增长55.7%；新开工亿元以上项目402个，增加224个。实施工业技改项目201个，完成投资146亿元，其中52个项目建成投产。招商引资成效明显。新签约投资亿元以上项目252个，协议总投资1862亿元。新开工亿元以上招商项目211个，到位资金342亿元。利用外资2.28亿美元，增长23%。联邦电工电缆产业园、华润热电、东阳光工业园、三宁己内酰胺、宜昌人福出口药业基地等一批重大项目开工建设，三新硅业、宜昌北500千伏输变电工程等一批重点项目建成。争取中央和省预算内投资项目1012个，到位资金突破20亿元。落实三峡对口支援项目资金92亿元。“十二五”重点项目储备入库5365个，总投资2.58万亿元。

　　（三）加快产业培育，经济结构进一步优化。工业转型步伐加快。规模以上工业企业完成增加值1190亿元，增长16.6%。化工产业产值突破1000亿元。磷化工、医药、电线电缆等7个产业集群进入省重点成长型产业集群。预计实现高新技术产业增加值260亿元，增长25%以上，占生产总值的比重提高0.8个百分点，达到10.4%。新增国家高新技术企业47家、国家技术创新示范企业3家。新增省级工程技术中心11家、省“两化融合”试点示范企业5家。新增主营业务收入过亿元企业76家、盈利过千万元企业42家。安琪酵母公司获“长江质量奖”。新增中国驰名商标6件，达到25件。

　　农村经济全面发展。农业生产再获丰收，粮食产量实现九连增。六大特色产业占农业总产值的比重达到78.7%，柑橘、茶叶、畜牧、蔬菜、水产总产再创历史新高。农业产业化深入推进。农产品加工产值达到1010亿元；新增规模以上农产品加工企业49家；稻花香500亿产业集群建设全面启动。农民专业合作社达到2793家，其中3家入选全国示范社。蔬菜、畜牧、水产等标准化生产示范基地建设加速推进。农业机械化作业综合水平提高到59.1%。实施土地开发整理项目17个，净增耕地面积2000公顷。全市“小农水”重点县实现全覆盖。除险加固小型水库66座。整治塘堰6783处。建设小水池4789处。新解决22.9万人饮水安全问题。治理水土流失面积80平方公里。完成县乡公路改造351公里、通村公路714公里。完成农村清洁能源入户工程3.5万户。

　　第三产业发展加快。完成三峡旅游度假区总体规划编制，部分项目开工建设。清江画廊成功创建国家5A景区。五峰地质公园升级为国家地质公园。新增全省旅游名村3个、5A级旅行社3家。全年接待游客2600万人次，旅游总收入190亿元，分别增长34.7%和34.5%。三峡现代物流中心建设有序推进。秭归翻坝物流园等物流园区建设加快。白洋作业区一期工程、茅坪作业区二期工程等港口建设项目顺利推进。新建乡镇商贸中心5家、县级配送中心1家，乡镇商贸中心和村级农家店累计达到1653家。新建村级综合服务社240家。发展“新网工程”连锁企业21家。文化产业不断壮大，增加值达到42.6亿元。当阳关公文化旅游城项目开工建设。金融业发展加快。组建了三峡农村商业银行。新成立保险业机构2家、村镇银行1家，开业运营小额贷款公司9家。

　　区域经济协调发展。县域经济占全市比重达到49%。宜都、夷陵、当阳、枝江继续保持全省县域经济发展先进行列，长阳县域经济综合指标考核进位速度全省第一。宜都高新区升级为省级高新区。点军、长阳先后被纳入国家可持续发展实验区。猇亭园区被纳入全国园区循环化改造示范试点。远安、秭归、兴山、五峰等山区县经济社会发展取得新成绩。13个乡镇进入全省百强乡镇行列，其中4个跻身20强。

　　（四）完善基础设施，城乡面貌进一步改善。交通建设投资超过80亿元。汉宜高铁通车营运，陆渔一级路基本建成。宜巴高速公路、保宜高速公路、紫云地方铁路建设顺利。宜张高速公路、庙嘴长江大桥、宜岳高速公路开工建设。创新理念、思路，强化现代化特大城市战略研究，完成城市总体规划修编。实施城区城建项目115个，完成投资30.5亿元。开工建设道路36条，总里程达89公里，其中13条道路竣工通车。生物产业园、白洋工业园、猇亭园区一批道路工程顺利推进。沙河整治工程、运河综合整治工程加快推进。猇亭、点军污水处理厂基本建成。城区亮化工程、户外广告整治和拆违控违工作有序推进。综合整治中心城区背街小巷24万平方米。城区菜市场改造基本完成。新增停车泊位1026个。文明城市创建成果进一步巩固，以较好成绩通过全国城市文明程度指数年度测评。顺利通过国家卫生城市复查。城市建设管理体制改革全面启动。新型城镇化进程加快，城镇化率提高到53%。城区新增建成区面积5平方公里。9个镇被列入全省中心镇，13个镇被列入全省特色镇，55个村被纳入全省新农村建设示范村。五峰新县城建设全面启动。生态建设成效明显。全市森林覆盖率达到65.7%，荣获“国家森林城市”称号。建成省级生态乡镇1个、生态村9个。实施节能技改项目20个、减排项目87个，节能减排年度任务全面完成。

　　（五）突出成果共享，民生福祉进一步提升。全年财政民生类支出205.5亿元，占地方公共财政预算支出总额的70%。新增城镇就业10万人，帮助失业人员和困难人员再就业5万人。城镇登记失业率降至3.1%。转移农村劳动力8.6万人。扶持7201人实现创业。社保扩面新增24.4万人，五项保险参保达到366.5万人次。城乡居民社会养老保险制度实现全覆盖，适龄人员参保缴费率达到95%。提高了新型农村合作医疗和城镇居民基本医疗保险补助标准、城乡低保补助标准、企业退休人员养老金标准。城区和部分县市建立了80岁以上高龄老人补贴制度。发放城乡困难群众社会救助资金4.7亿元。建成农村互助养老中心87个。保障性住房开工2.96万套，竣工1.76万套，新增廉租房租赁补贴4000户。完成农村危房改造1.7万户。新建城郊蔬菜基地1万亩。市场物价总体平稳。长阳、五峰、秭归进入国家武陵山片区扶贫攻坚规划。全市当年稳定脱贫3.67万人。

　　财政性教育支出增长31.7%。加快实施学前教育“三年行动计划”，新建、改扩建幼儿园30所，学前教育管理进一步规范。全市所有县市区均通过省级义务教育均衡发展合格县市区考评。创建现代化初中学校41所、小学70所。城区教育体制改革正式启动。宜昌职教园建设进展顺利。累计竣工校舍安全工程594个。“学生营养工程”和“农村义务教育学生营养改善计划”惠及学生45万名。资助贫困学生36.8万人次、1.9亿元。加大人才工作力度，引进国家“千人计划”专家2人、省“百人计划”专家5人。医药卫生体制改革深入推进。全民医保、国家基本药物制度和基层医疗机构综合改革进一步深化，城乡居民医保参保率达到98.1%，基本药物零售价下降31.1%。当阳、枝江公立医院综合改革试点进展顺利。公共文化服务体系进一步完善，市群艺馆新馆等文化重点项目建设加快推进。文化信息资源共享、乡镇综合文化站、村文化室、农家书屋等文化惠民工程实现全覆盖。群艺馆、文化馆、公共图书馆全面实现免费开放。《巴土恋歌》获全国“五个一工程”奖。成功举办屈原故里端午文化节、第二届中国长江钢琴音乐节等重大文化活动。抢救发掘了一批西周中晚期青铜器珍贵文物。文化体制改革顺利推进。全民健身活动广泛开展，竞技体育水平显著提高，宜昌籍选手赵芸蕾勇夺2枚奥运金牌，实现宜昌奥运金牌“零”的突破。深化“一本三化”社会服务管理创新，经验在全国推广。“六五”普法工作全面启动，公民法律意识进一步增强。社会管理综合治理成效明显，群众安全感和治安满意度稳步上升。食品安全放心城市创建工作全面启动。全市安全生产形势总体稳定。

　　（六）改进政务服务，行政效能进一步提高。取消市级行政审批事项20项，下放9项。加强电子政务建设，行政效率不断提高。坚持依法行政、从严治政，政府执行力和公信力不断增强。认真听取人大代表、政协委员意见和建议，按期办复人大代表议案建议320件、政协委员提案428件，办结率100%。行政监察和审计监督更加有力，治庸问责深入推进，党风廉政建设责任制全面落实。

　　服务三峡工程运行成效明显，库坝区管理安全有序。援藏援疆工作深入开展。国防动员、民兵预备役建设和双拥工作不断加强，连续五届荣获“全国双拥模范城”称号。出生人口性别比保持正常，人口和计划生育工作继续保持领先水平。对台、侨务、外事、保密、信访、档案、方志、统计、气象、人防、民族宗教、防震减灾等各项工作都取得新的成绩，妇女儿童、青少年、关心下一代、老龄、残疾人等各项事业全面发展。

　　各位代表，这些成绩和进步，是在宏观环境复杂严峻、发展制约因素不断增多的情况下取得的，来之十分不易，凝聚着全市人民的心血汗水，见证着方方面面的大力支持。在此，我代表市人民政府，向全市广大干部群众，向全体人大代表、政协委员和各民主党派、工商联、各人民团体、无党派人士，向驻宜解放军指战员、武警官兵，向在宜中省单位和广大外来投资者，向所有关心支持宜昌发展的海内外朋友，表示崇高的敬意和衷心的感谢！

　　我们清醒地看到，国内外经济运行环境依然复杂，中部地区和长江沿线城市发展竞争更加激烈，宜昌发展仍面临不少矛盾和挑战。主要是经济总量和规模不够大；少数企业运行十分困难；自主创新能力不足，产业结构层次不高，战略性新兴产业和现代服务业相对薄弱；制约科学发展的体制机制障碍仍然存在；社会保障水平与民生需求还有一定差距；行政效能有待进一步提高。对此，我们一定高度重视，采取有力措施加以解决。

　　二、2013年工作重点

　　刚刚召开的党的十八大，对推进中国特色社会主义事业作出“五位一体”总体部署，科学描绘了全面建成小康社会的宏伟蓝图。省第十次党代会，要求宜昌加快建设省域副中心城市、长江中上游区域性中心城市、世界水电旅游名城，加快建设现代化特大城市，宜昌已站在新的历史起点上，我们对宜昌的发展充满信心。当前，宜昌正处于跨越发展的黄金十年、关键五年，加快发展的有利因素很多。国家促进中部崛起战略深入实施，三峡后续工作规划、武陵山区域发展规划和新一轮扶贫攻坚规划全面推进，国际国内产业转移步伐加快。长江中游城市群正在成为全国经济发展重要增长极。湖北加快构建中部崛起重要战略支点，全面实施“一元多层次”战略体系，这些重大机遇正在宜昌产生叠加效应。近年来，一批重大项目陆续落户宜昌，产业发展形成多极增长、交替领跑的良好格局，为加快发展提供了重要支撑。特别是去年以来，省委进一步调整宜昌领导体制、加强宜昌的领导力量，市委动员全市上下深入开展“解放思想、激情跨越”学习讨论活动，以思想大解放推动发展新跨越，必将激发全市人民加快建设现代化特大城市的强大动力。

　　2013年是全面贯彻落实十八大精神的开局之年，实施“十二五”规划承前启后的关键一年。做好今年工作，对于推动经济社会跨越式发展，具有十分重要的意义。我们将始终坚持以邓小平理论、“三个代表”重要思想、科学发展观为指导，全面贯彻党的十八大精神，深入解放思想，深化改革开放，创新体制机制，转变发展方式，促进工业化、信息化、城镇化、农业现代化同步发展，稳中求进，竞进提质，着力构建现代产业发展新体系，着力推进新型城镇化，加快推进文化建设，加快生态文明建设，加快提升城市创新能力、综合实力和竞争力，切实保障和改善民生，坚定不移地向现代化特大城市迈进。

　　今年，全市经济社会发展主要预期目标是：生产总值增长12.5%，全社会固定资产投资增长25%，社会消费品零售总额增长16%，地方公共财政预算收入增长14%，外贸出口增长10%，城镇居民人均可支配收入、农村居民人均纯收入增长与经济发展同步，城镇登记失业率控制在4.2%以内，物价水平基本稳定，全面完成省政府下达的节能减排任务，努力保持经济快速健康发展、社会和谐稳定。制定这些目标，充分考虑了宜昌发展的基础条件、区域竞争态势和省委省政府“三个三分之一”经济战略布局的要求，是积极进取并切实可行的。围绕这些目标，市政府将重点抓好五个方面的工作。

　　（一）坚定不移地加快转型升级，推动经济发展新跨越

　　牢固树立产业第一理念，转变经济发展方式，加快推进工业做大做强、服务业快速壮大、特色农业提质增效，提高经济发展的质量和效益，全面提升产业竞争力和综合实力。

　　大力实施创新驱动战略。把创新驱动摆在宜昌发展全局的核心位置，不断推动体制机制创新、领导经济工作的方式方法创新，发挥科技创新的战略支撑作用，激发全社会的创新创造活力，使经济发展更多依靠科技进步、劳动者素质提高、管理创新驱动。加大财政引导性资金投入力度，坚持以企业为主体、产业为核心、人才为支撑、环境为保障，完善区域创新体系，推进国家创新型试点城市创建工作。创新人才工作体制机制，创优人才环境，加大人才工作投入和政策落实力度，建设“人才强市改革试验区”。紧扣全市重点产业和重要领域需求，大力引进掌握领先技术、引领产业发展的创新创业人才及其他各类高层次人才，力争新引进培养2至3名中央“千人计划”、省“百人计划”人选。引导创新要素向优势企业聚集，加大科技型中小企业扶持培育力度，确保新增国家高新技术企业40家以上，高新技术产业产值、增加值增长25%以上。突出科技成果转化应用，加快高新技术成果产业化进程。加强科技企业孵化器等平台建设，大力推进自主创新，使更多的“宜昌制造”转变为“宜昌创造”。新增省级以上工程技术中心6家，新建市级工程技术中心、重点实验室10家。深入推进知识产权战略，抓好专利申请工作，确保全市专利申请量和授权量增长10%以上。

　　培育工业经济竞争优势。坚定不移地走新型工业化道路，加强产业政策引导，推动主导产业延伸产业链、向价值链高端攀升，壮大产业规模，提升产业发展水平。通过3至5年努力，把宜昌建成世界最大的水电能源基地，全国最大的磷化工生产基地，全国重要的食品饮料生产基地、生物医药生产基地、先进装备制造基地、新材料生产基地和循环经济示范基地。确保今年规模以上工业增加值增长16%以上，化工产业产值突破1400亿元、先进装备制造产业突破900亿元、食品饮料产业突破800亿元、新材料产业突破500亿元。出台有针对性的政策措施，支持龙头企业发展、重点项目建设，大力培育汽车和船舶制造、生物医药、新材料等一批战略性新兴产业集群，尽快形成新的产业支柱。加快实施重大技术改造项目，推进建材、纺织等传统产业转型发展。推动信息化与工业化深度融合，培育一批“两化融合”示范企业。强化市场主体培育。帮助150家骨干企业制定发展规划，实施优强企业倍增工程。统筹使用产业发展基金、新型工业化资金，采取阶段参股、风险投入、贷款贴息、短期调度资金支持等方式，支持企业技术改造、兼并重组、发行债券、上市融资。建立中小企业成长工程后备库，抓好“小进规”工作，力争新增规模以上工业企业100家。加大精品名牌培育力度，引导企业推行卓越绩效管理，积极争创全国质量强市示范城市。加强生产要素协调，强化工业经济运行监测服务，保障企业正常生产经营。

　　突破性发展现代服务业。进一步树立“大旅游”理念，整合旅游资源，促进文化旅游深度融合，创新旅游发展模式，加快建设国际知名、国内一流的旅游目的地城市。依托“两坝一峡”区域，进一步创新体制机制，打破行政区划界限，理顺产权关系，实现合作共赢，加快长江三峡国家级旅游度假区建设。全面深化与长江三峡集团、葛洲坝集团和鄂西圈投公司的战略合作，强化政策支持，吸引社会投资，推进三峡游轮中心、平湖半岛等一批重点项目建设。以清江画廊5A景区为龙头，加强景区标准化建设，提高景区服务水平，推动与柴埠溪等景区整体营销、联动发展，着力打造清江民俗风情旅游区。发掘宜昌历史文化资源，彰显宜昌现代文化特色，着力打造文化旅游主题景区。积极创建国家级地质公园。规划建设地质馆、茶博馆等一批特色博物馆，展示宜昌城市魅力。办好第四届长江三峡国际旅游节和第九届中国国际民间艺术节。确保全年接待游客3000万人次，旅游总收入240亿元。加快建设三峡现代物流中心。抓紧编制重点物流园区建设详规。依托机场、港口和火车站，突出抓好港口综合型、货运枢纽型、市场配套型物流园建设，大力发展临港、临空、临铁产业。研究制定鼓励物流业发展的相关政策，支持发展第三方物流，培育专业化物流企业，大力引进国际国内有实力的物流公司。加快建设区域性科教文化中心。坚持政府引导和市场推动相结合，促进文化与科技融合，发展壮大出版、印刷、广告、演艺娱乐等传统文化产业，加快培育文化创意、数字动漫、文化会展等新兴文化产业。加大政策扶持力度，加强文化产业招商引资，积极培育文化市场主体，打造具有地域和民族特色的文化产业集群。力争通过3至5年努力，全市文化市场主体突破1万家，产值过亿元文化企业达到20家以上，文化产业增加值占生产总值比重达到6%。积极发展信息服务、科技服务、商务服务业，推进信息技术在政务商务、生产生活、社会管理等领域的深度应用，大力发展移动互联网、云计算、物联网等新兴业态，着力引进一批服务外包龙头企业，推进服务外包产业发展。优化商贸布局，丰富商贸业态，创新商业模式，新增限额以上商贸企业150家。积极争创国家电子商务示范城市。新建村级综合服务社200家。大力发展总部经济和楼宇经济。稳定发展房地产业。优化金融生态环境，加快建设区域性金融中心。着力引进各类金融机构到宜昌设立区域性总部。积极培育小额贷款公司，力争新成立8家。设立村镇银行3家。完善激励政策，积极引进培育创业风险投资等金融实体。支持企业上市，鼓励企业进入场外市场进行股权交易。力争今年实现1家企业上市，新增2家企业进入报备辅导期。

　　大力发展现代特色农业。围绕建设全国有影响的特色农产品生产大市、中部地区农产品加工强市、全省现代农业示范市目标，进一步健全现代农业产业体系，确保今年农产品加工产值过1200亿、农村居民人均纯收入突破9000元。实行最严格的耕地保护制度和节约集约用地制度，改造高产农田52万亩。全面提升柑橘基地建设、保鲜贮藏、产品加工、市场营销水平，推进产业升级，使柑橘成为农民增收的优势产业。积极创新茶产业发展模式，支持发展精深加工，加快建设现代茶叶生产技术集成基地，努力跻身全国最有影响力的茶产区行列。以发展城郊蔬菜和高山反季节蔬菜为重点，推进品种结构调整，培育加工龙头企业，加强蔬菜品牌建设。继续加大畜牧、水产、魔芋、中药材、食用菌、木本油料、苗木花卉等特色产业发展力度，实现特色农业提质增效。积极发展都市农业、乡村休闲农业。以示范基地建设为抓手，大力实施标准化生产，着力提升优质农产品生产水平。加快推进农产品精深加工，着力构建农业产业化经营体系。加大政策扶持力度，组织实施百亿企业成长工程、农产品加工示范园区创建工程、名优品牌创建工程。规范发展农民专业合作社，提高农民组织化程度。加强农业科技创新服务体系建设，支持龙头企业打造农业科技产业链，提升农产品科技含量和规模化水平。扎实做好农产品销售工作，开展国家农产品现代流通试点，推进农商农超对接，鼓励企业开拓国内外市场。

　　加快园区和项目建设步伐。把园区作为产业发展的第一平台，坚持产城共育，坚持先规划后建设、先生态后生产、先地下后地上、先配套后招商，加快推进宜昌高新区园区建设。完善高新区经济社会管理、投融资、财税分配等体制机制，创新开发模式，提高园区运转效率。集中连片开发生物产业园、白洋沙湾片区和田家河片区，强力推进道路、管网、绿化等配套工程建设。灵活运用市场化融资方式，引导民间资本投资园区开发，参与基础设施建设，改善项目落地条件。支持县市区建设专业化特色园区。抓住中央实施积极财政政策的机遇，组织推动各方力量，有效对接国家、省发展规划，精心策划一批重大产业项目、科技文化项目、城市建设项目和基础设施项目，不断充实项目储备库。加强项目跟踪服务，扎实推进金帆达化工新材料、南玻精细玻璃和超薄电子玻璃、中兴汽车、湖北化肥乙二醇、三峡物流园等重点项目建设，确保固定资产投资突破2000亿元。进一步完善重大项目建设工作机制，强化项目督办检查，切实提高供地、环评、规划等环节的服务效率，切实增强项目建设实效，促进一批重大项目签约落地、一批重大项目开工建设、一批重大项目建成投产。创新土地利用机制，开发利用荒山荒地，盘活存量土地，提高土地利用效率，确保项目用地需求。

　　（二）坚定不移地推进新型城镇化，奠定特大城市新格局

　　坚持高起点规划、高标准建设、高水平管理、高效益经营，创新城市发展理念，着力推进宜昌新区建设，加快建设既大又强、特优特美的现代化特大城市，全面提升城市产业集聚功能、综合服务功能、辐射带动功能，实现城市规模和质量协调发展。

全面推进宜昌新区建设。实施跨江发展、两岸并进，加快东站片区和点军片区等重点区域开发建设，全力打造规划面积200平方公里、建设用地100平方公里的宜昌新区。完善新区规划和实施方案，制定分区规划和片区开发计划，产城共育，统筹推进，打造宜昌城市新标志、经济发展新平台、群众生活新天地。力争三年完成新区建设投资1000亿元，建成20平方公里核心区，实现100平方公里基础设施覆盖，建成骨干道路160公里，新建1000万平方米房屋，建设6平方公里的公共绿地。今年完成投资240亿元以上，实施142个城市建设项目。以道路建设引导城市开发，统筹规划以轨道交通、公交BRT为重点的公共交通体系、慢行系统、绿道系统及微循环系统，重点建设跨区、跨组团、内外通联的快速路及主干道。开工建设东山四路、东岳二路等快速骨干道路。按照大分散、小集中的原则，合理布局行政、体育、科教文化、交通物流、商务商贸等公共设施，开工建设新博物馆、规划展览馆、奥林匹克运动中心，启动行政服务中心、大剧院、美术馆、音乐厅等项目前期工作。坚持“终极产品、一步到位”理念，努力打造百年建筑、个性城市。

　　加快完善新型城镇体系。加快编制宜昌城乡全域规划，推进全市产业布局、基础设施、社会事业、生态保护、公共服务管理统筹发展。按照“集约发展、布局协调、结构合理”的总体要求，积极构建中心城区和宜都、当阳、枝江、秭归、长阳“一主五副”城镇结构，形成长江和香溪河、沮漳河、清江、渔洋河流域“一带四廊”城镇发展空间格局。宜都、当阳、枝江按照20万人口中等城市的标准，秭归、兴山、远安、长阳、五峰等县城按不低于5万人标准，统筹规划，扩容提质。支持县域中心镇发展，每个县市建成1至3个综合实力突出、布局合理、规模适度、功能健全、特色鲜明的中心镇或特色镇。构建特大城市、中等城市、小城市和小城镇科学布局、协调发展的宜昌城市格局，着力提高城镇化质量。加快市域交通路网建设，积极构建“大宜昌”一小时交通圈。加快建设庙嘴长江大桥，开工建设香溪长江大桥，加快推进白洋长江大桥前期工作。加快推进宜岳、宜张高速公路建设，确保宜巴高速宜昌段基本建成，保宜高速宜昌段路基贯通。开工建设伍家岗至龙泉、龙舟坪至五眼泉等一级公路。加快推进318国道万城大桥至云池段一级公路建设。加快建设三峡枢纽港重点港区。加快三峡机场改造进程。

　　深入推进新农村建设。编制和实施县域村镇体系规划，以中心村为重点，以新型农村社区为抓手，抓好村庄建设和农村住房建设，促进土地节约、资源共享，提高农村基础设施和公共服务水平。确保3至5年内，实现市郊和建制镇周边新型农村社区建设全覆盖，建成100个示范宜居村庄。深入开展“万名干部进万村洁万家”活动。加大对城中村、城乡结合部改造力度，抓紧编制并严格执行城乡生活垃圾收运处理、城镇污水处理、农贸市场、停车场等专项规划。实施“清洁乡村”工程，扎实开展农村环境综合整治，实现农村环境净化、道路硬化、房屋美化、路街亮化、村庄绿化和文化体育设施配套。建立城乡一体的污染防治监控管理体系，探索建立和完善城乡一体的生活垃圾和污水处理长效机制，建立健全县乡村三级环卫管理体制。完成农村清洁能源入户工程3万户。解决农村10万人以上的饮水安全问题。改造末级渠系700公里，整治堰塘、建设抗旱水池3000口。新建100公里二级公路，建设300公里县乡公路、1000公里村级公路。

　　努力打造美丽宜昌。坚持把生态文明建设放在更加突出的位置，贯穿到城市规划、建设和管理每个环节，体现到城市空间结构、产业布局、生产方式和生活方式各个方面，促进生产空间集约高效，生活空间宜居适度，生态空间山清水秀。抓紧编制城市环境总体规划，积极开展环境污染防治、生态区域保护等专题研究，加强生态保护和建设，提高城市可持续发展能力，实现人与自然和谐发展。巩固国家森林城市创建成果，加强森林抚育，提高林分质量，新建集中连片千亩造林基地10个，植树造林20万亩。加快推进西坝污水收集及输送工程、花艳污水处理厂二期等环保项目建设。关闭美联、安能热电厂，进一步改善城区大气环境。加强长江岸线保护与开发。实施磨基山公园、东山公园改造工程，推进求雨台公园、卷桥河公园等生态工程。利用运河等城中水系，规划建设更多的城市水景。实施最严格的水资源保护制度，建设节水型社会。加快运河、黄柏河、沮漳河等流域综合治理，加快三峡库区水污染防治项目建设进程。完成治理水土流失面积50平方公里。加强集中式饮用水水源地保护。抓好国家再生资源回收体系建设试点。进一步做好节能减排工作。加强大气环境综合监测。推进城区机动车尾气排放“黄绿标”管理。坚持依法管理、科学管理、精细管理，塑造“洁、绿、亮、美、静、畅”的城市环境，巩固和扩大全国文明城市创建成果。

　　（三）坚定不移地推进改革开放，增强跨越发展新动力

　　坚决破除影响和制约科学发展的思想观念和体制机制弊端，构建科学规范、运行有效的制度体系，打造更具活力、更加开放的发展环境，形成充分调动市内外最广泛资源推进宜昌跨越式发展的新格局。

　　深化重点领域改革。围绕建设现代化特大城市目标，抓住关键环节，创新体制机制。推进城区财政体制、教育体制、城市建设管理体制改革。创新投融资体制机制。创新农村土地管理、社会管理体制机制，统筹推进城乡一体化发展。筹建农村综合产权交易中心。优化民营经济发展环境，降低准入门槛，加强服务引导，鼓励和吸引更多社会资本进入公用设施建设、社会事业发展等领域。深化行政审批制度改革，创新行政服务管理方式方法，实施行政审批流程再造，推进行政审批服务质量管理体系认证，打造全国审批事项最少、行政效率最高、办事成本最低的政务服务环境，积极建设效能宜昌。加快完善文化管理体制和文化生产经营机制，健全国有文化资产管理体制，继续做好市直国有文艺院团改革工作。深化医药卫生体制改革，力争在城乡居民大病医疗保险、公立医院改革等方面取得新进展。完善公务员管理机制，稳妥推进事业单位分类改革。

　　提升招商引资水平。完善招商机制，创新招商引资方式方法。加强招商队伍建设，形成招商合力。着力拓展招商信息渠道，实现信息共享，统筹利用，努力在城市建设、文化旅游、高端物流、电子信息、生物医药、汽车及零配件制造、新能源、新材料、农产品深加工等领域实现招商引资新突破。加强项目科学论证，提高招商引资质量和效益。抢抓国际资本与沿海产业转移机遇，提升利用外资水平，吸引跨国公司、知名企业在宜设立区域总部、研发制造中心等功能性机构。加强与中省企业战略合作，支持在宜中省企业做大做强。争取三峡对口支援和经济协作项目资金60亿元。力争新开工亿元以上招商项目265个，全市招商引资实际到位资金900亿元。

　　扩大对内对外开放。敢开放、真开放、先开放、全开放，增强合作意识，实现共赢发展，营造开放包容、大气开明的人文环境，重商亲商、懂商敬商的投资环境，诚实守信、优质高效的服务环境，舒适便捷、宜居宜旅的生活环境。推进与长江中游城市群融合，加强与珠三角、长三角地区和成渝城市圈的有效对接，主动承接国际国内产业转移，积极参与区域产业分工合作与交流，打造内陆开放高地。积极推进保税仓库和水运、航空口岸建设，改善物流、交通和口岸大通关条件，促进贸易投资便利化。鼓励航空、港口、高铁积极开辟新的航线、班次。推进宜昌港与武汉新港、南通港、重庆港的全面对接。加强出口基地建设，支持企业建设境外营销网络，积极开拓新兴市场，推动出口主体和出口市场多元化。创新“走出去”和“引进来”方式，鼓励有条件的企业对外直接投资，支持企业引进战略投资、高新技术、关键设备和先进管理经验。

　　（四）坚定不移地保障和改善民生，实现社会建设新进步

　　积极顺应人民群众过上更加美好生活的新期待，加大公共财政投入力度，加快形成政府主导、覆盖城乡、可持续的基本公共服务体系，着力构建充分就业、体面劳动、合理分配、幸福生活的共建共享新格局，让改革发展成果更多地惠及全市人民。

增加城乡居民收入。认真落实国家、省增加城乡居民收入的一系列制度安排，全面实施就业提升、创业富民、政策普惠等工程，不断提高居民工资性、经营性、转移性和财产性收入。严格执行最低工资标准，深入推进工资集体协商，着力完善工资正常增长机制和支付保障机制，保护劳动者合法权益。突出创业致富，深入推进全民创业，鼓励“二次创业”。支持投资服务与理财机构发展，鼓励居民依法开展投资经营活动。扎实推进新一轮扶贫开发，大力支持武陵山片区、秦巴山片区贫困县加快发展，实施86个村整村扶贫攻坚，扶贫搬迁1120户，稳定脱贫3.8万人。抓好大中型水库移民后期扶持工作，促进移民安稳致富。推进农村土地承包经营权健康有序流转，提高农民土地增值收益。充分发挥政府价格调节基金作用，确保城乡困难群众基本生活水平不因物价上涨而降低。

　　加强就业和社保工作。实施就业优先战略和更加积极的就业政策，多渠道开发就业岗位，强化就业服务，突出解决好困难群体就业，鼓励支持更多的劳动者自主创业、自谋职业和灵活就业，最大限度增加就业容量。开展各类职业技术培训，加快紧缺性技能人才培养，提高劳动者职业素质和就业创业能力，促进更高质量就业。确保新增城镇就业7.7万人，下岗失业人员和困难人员再就业2.75万人，农村劳动力转移就业7.5万人。强化劳动监察与维权，努力构建和谐劳动关系。加强社会保障体系建设，稳步扩大社会保险覆盖面，确保扩面15万人次。认真做好不同群体社保制度的衔接转换。全面推进城乡居民社会养老保险。巩固完善新型农村合作医疗制度。加大保障性住房建设力度，开工建设保障性安居房2.5万套。严格监管，促进保障性住房公开、公平、公正分配。支持养老服务机构建设，健全社会和居家养老服务网络。全面实施80岁以上高龄老人补贴制度。新改造农村危房1.4万户。提供残疾人康复服务1.7万人次，免费提供辅助器具5500套。加强特殊群体社会救助与保障。

　　推进文化建设提速。把文化建设摆在更加重要的位置，全面增强宜昌文化创造活力，不断提升宜昌文化整体实力和竞争力。积极培育社会主义核心价值观，大力弘扬宜昌城市精神，广泛开展扶危济困、邻里互助、爱国卫生等志愿服务，推动学雷锋活动、学习宣传道德模范常态化，不断提高市民文明素养。加强和改进未成年人思想道德教育。坚持面向基层、服务群众，加快市县乡村公共文化设施建设，推进城乡文化一体化发展。完善广播电视“村村通”工程，改造一批高山无线发射平台。继续推动公共文化服务设施向社会免费开放，广泛开展文化下乡、文化进社区、全民阅读等群众性文化活动，丰富城乡群众精神文化生活。积极创建国家公共文化服务体系示范区。加强文物和非物质文化遗产保护，做好第一次全国可移动文物普查工作。发展哲学社会科学、广播电视电影、新闻出版、文学艺术事业。鼓励文艺创作，推出一批文艺精品。加强主流文化网络传播，扩大对外文化交流，提升宜昌文化软实力。

　　加快社会事业发展。加大教育投入，进一步提高全市教育支出占公共财政支出比重。全面完成学前教育“三年行动计划”，确保学前三年入园率达到80%。大力推进教育信息化和现代化学校创建活动。加快宜昌职教园建设，抓好市青少年示范性综合实践基地建设，全面完成校舍安全工程建设任务。完善义务教育资源共建共享和对口交流支援机制。支持三峡大学等高等院校发展。鼓励和规范社会力量办学。免除中等职业教育农村学生学费。积极推动进城务工人员子女平等接受教育。办好特殊教育。重视继续教育，建设学习型城市。巩固国家卫生城市创建成果，推进“健康宜昌”创建活动，加快建设区域性医疗卫生中心，不断提高人民群众健康水平。健全农村三级医疗卫生服务网络，完善城市社区卫生服务体系，努力让群众享有安全优质、方便价廉的基本医疗服务。加快推进市中心医院、市一医院、市妇女儿童医院等基础设施建设，支持重点医院争优创名。加强卫生信息化建设。建立和谐互信医患关系。举办市第四届运动会。实施全民健身计划，新建省级农民体育健身工程90个、市级全民健身路径35条、村级体育设施180个，促进群众体育和竞技体育全面发展。普及科学知识，弘扬科学精神，提高群众科学素养。突出利益导向，统筹解决人口问题，促进人口长期均衡发展。实现免费孕前优生健康检查全覆盖。加强统计基础工作，健全统计调查体系，扎实做好全国第三次经济普查。认真实施妇女儿童发展规划。积极支持工会、共青团、妇联等参与经济社会管理和公共服务。扎实做好民族团结进步工作和援藏援疆工作。依法管理宗教事务。统筹推进机构编制、档案、方志、慈善、外事、侨务、港澳、对台、保密、气象、地震等各项工作。大力支持驻宜解放军和武警部队现代化建设，加强国防后备力量建设，提升国防动员、国防教育、民兵预备役和人民防空水平，做好优抚安置、军休服务和军供保障工作，巩固和发展军政军民团结，再创“全国双拥模范城”。

创新社会服务管理。加强社会建设，建立健全社会管理体制机制、基本公共服务体系、现代社会组织体制。坚持民生优先、服务为先、基层在先，推动网格化管理、信息化支撑、全程化服务体系向县市延伸，不断提升社会管理水平。认真做好群众来信办理和来访接待，妥善解决群众合理诉求。深入推进社会稳定风险评估，不断健全矛盾纠纷排查调处机制，有效预防、处置各种突发公共事件和风险。加强社会管理综合治理，完善流动人口和特殊人群管理服务，健全立体化社会治安防控体系，强化司法基本保障，依法防范和惩治违法犯罪活动，致力打造平安宜昌，争创全国社会管理综合治理“长安杯”。加强法律服务、法律援助和普法宣传教育，培育法治文化，弘扬法治精神，加快建设法治宜昌。切实维护三峡坝区社会稳定和库区蓄水安全，全力服务三峡工程安全运行。加快社区公共服务能力建设。加强粮油市场建设和管理，推进国家肉类蔬菜流通追溯体系建设试点，完善食品药品监管体制机制，提高监管能力，加快创建食品安全放心城市。加强应急管理与防灾减灾体系建设。全面落实安全生产责任制，继续强化交通、矿山、化工、建筑、消防等重点行业、重点领域安全隐患排查和专项整治，坚决遏制重特大事故发生。

　　（五）坚定不移地推进勤政善政，塑造人民政府新形象

　　深入解放思想，勇于改革创新，始终勤勤恳恳、兢兢业业地服务人民、推动发展，努力建设为民、务实、清廉的服务型政府，促进政府工作不断进步。

　　激情奋进，保持一流的精神状态。紧紧围绕宜昌发展的宏伟蓝图和目标，着力提升精神区位，始终以旺盛饱满的热情、奋发有为的豪情对待事业，切实把精力和智慧集中到应对挑战、谋划发展、改善民生、促进和谐上，构建强大的发展气场，以干部群众的精神高位赢得宜昌发展高位。强化担当意识、增强担当勇气、提升担当能力，敢于担责，敢于担难，敢于担险。大力倡导敢想敢试、敢闯敢冒的进取精神，以更大的胆识和气魄推进改革与发展，不断增创新优势、实现新突破，努力赢得发展主动权。

追求卓越，树立一流的工作标准。按照科学发展跨越式发展的要求，以更加开放的视野、更加科学的思维、更加有效的办法，谋划和推进各项工作。深入学习党的十八大精神，准确把握中国特色社会主义理论体系本质内涵，用马克思主义中国化最新成果武装头脑、指导实践。坚持向书本、实践和群众学习，不断掌握新知识，积累新经验，增长新本领。认真学习借鉴发达地区的思路、理念、经验，切实将外出“三省八市”学习考察和“解放思想、激情跨越”大讨论中形成的思想成果，转化为履行职责的本领、转化为深化改革的思路、转化为推动发展的实践，不断加快跨越赶超步伐。强化调查研究，注重战略思考、超前谋划，增强工作的主动性和前瞻性，努力提高复杂形势下驾驭经济工作的能力。着力完善行政决策机制，确保决策科学化、民主化、法治化。全面推进依法行政，积极运用法治思维和法治方式深化改革、推动发展、化解矛盾、维护稳定。深入推进政务公开，自觉接受人大、政协、群众和社会的全方位监督，高标准、高质量办理好各类议案、建议、提案和民生事项。

　　务实高效，发扬一流的工作作风。大力弘扬求真务实精神，重实干、讲实效，聚精会神、心无旁骛地抓发展、抓落实，多做打基础、管长远、惠民生的实事好事，实现没有水分的快速增长。认真学习中央政治局“八项规定”，深入开展以为民务实清廉为主要内容的群众路线教育实践活动，大力改进工作作风，密切联系群众。下决心精简会议、文件，摆脱繁琐事务，带头深入基层，深入到条件差、困难多、工作进展慢的地方解决问题、服务发展，努力让企业满意、群众满意。加强政风行风建设，加快推进电子政务，努力建设智能政府、效能政府，全面提升政务服务效率和质量。加大决策执行和工作落实力度，定下来的事情雷厉风行、马上就办。强化效能监察、审计监督和治庸问责，推进工程建设、土地、产权、政府采购等公共资源交易规范管理，加强对重要工作、重点项目、重大工程的跟踪督查，切实防止行政不作为、慢作为、乱作为，确保行政规范、政令畅通，为各类市场主体营造放心投资、安心发展的良好环境。提倡艰苦奋斗，厉行勤俭节约，力戒铺张浪费。全面落实党风廉政建设责任制和领导干部廉洁自律各项规定，加强干部队伍纯洁性建设。

　　开拓创新，争创一流的工作成效。始终把开拓创新作为促进科学发展跨越式发展的关键之举，作为战胜各种困难挑战的制胜之道，贯穿于政府工作全过程，让发展思路、工作部署和政策措施更加符合实际、顺应民意、富有创新。营造勇于开拓、善于创新的氛围，不断推进理念、思路、体制、机制等方面的创新，坚持用创新的思维、改革的思路、市场的办法破解发展难题，激活发展活力，增强发展优势。巩固创先争优活动成果，鼓励和支持全市各地各部门用好用活各项试点赋予的“先行先试权”，不断推出在全省乃至全国具有示范意义的创新之作，努力在发展目标、发展模式、发展成效上争创一流，切实让推进发展的各项工作经得起实践检验、群众检验和历史检验。

　　各位代表！宏伟蓝图引领着我们，庄严使命激励着我们，美好未来召唤着我们。让我们高举中国特色社会主义伟大旗帜，认真贯彻落实党的十八大精神，在省委省政府和市委的坚强领导下，提神增劲，凝心聚力，倍道兼程，锐意进取，为把宜昌建成省域副中心城市、长江中上游区域性中心城市、世界水电旅游名城和现代化特大城市而努力奋斗！
